# Polyphenol-Rich *Citrullus lanatus* Rind Extract Mitigates Doxorubicin-Induced Cardiotoxicity: HPLC Profiling and In Vivo Evaluation

**DOI:** 10.3390/pharmaceutics17111469

**Published:** 2025-11-14

**Authors:** Bader Alsuwayt

**Affiliations:** Department of Pharmacy Practice, College of Pharmacy, University of Hafr Al Batin, Hafr Al Batin 39524, Saudi Arabia; balsuwayt@uhb.edu.sa

**Keywords:** polyphenols, hesperetin, hydroxy benzoic acid, doxorubicin, cardiovascular risk, oxidative stress, tumor necrosis factor-α

## Abstract

**Background/Objectives**: Cardiovascular diseases (CVDs) remain a major cause of mortality globally, driven in part by oxidative stress and inflammation. The present study investigated the polyphenolic composition and cardioprotective potential of polyphenol-rich *Citrullus lanatus* (PRCL) rind extract against doxorubicin-induced cardiotoxicity in rats; **Methods:** High-performance liquid chromatography (HPLC) was employed to identify and quantify the major bioactive compounds present in the extract. Total 30 healthy male *Wistar Kyoto* rats were recruited and divided into 6 groups and various cardiovascular markers and antioxidant were measured in vivo and in vitro methods; **Results:** Ethanolic extraction of *Citrullus lanatus* rind yielded 19.58 g extract per 100 g of dry plant material. HPLC analysis identified five phenolic acids, i.e., gallic acid, *p*-hydroxybenzoic acid (PHBA), chlorogenic acid, caffeic acid, and vanillic acid, and two flavonoids, i.e., catechin and hesperetin, with PHBA (163.66 mg/g of extract) being the most abundant. Total phenolic and flavonoid content was determined to be 35.6 mg GAE/g and 12.8 mg CE/g, respectively. In vitro antioxidant assays showed moderate free radical scavenging, reducing power, and 86.9% inhibition of linoleic acid peroxidation. In vivo, Wistar rats were treated with doxorubicin (10 mg/kg) to induce cardiotoxicity, followed by PRCL extract administration (21 days at 250 and 500 mg/kg/day). The extract significantly improved body weight, serum lipid profile, and reduced cardiovascular risk indices. Antioxidant biomarkers (SOD, CAT, GPx, GSH) were restored, while lipid peroxidation (MDA) and inflammatory cytokines (TNF-α, IL-6) were significantly reduced in treated groups. The 500 mg/kg dose demonstrated superior efficacy, comparable to the standard quercetin group. Histopathological examination revealed notable protection of cardiac tissue architecture in the high-dose PRCL-500 group; **Conclusions:** These findings suggest that PRCL rind extract contains potent compounds having antioxidant and cardioprotective properties and may be used as a natural therapeutic agent against cardiotoxicity.

## 1. Introduction

Cardiovascular diseases (CVDs) are chronic conditions influenced by various risk factors, which include atherosclerosis, myocardial infarction, stroke, hypertension, heart failure, hyperglycemia, diabetes, obesity, and kidney dysfunction [1,2,3]. Among these factors, ischemic heart diseases (IHDs), heart failure, stroke and hypertension contributed 82% of the global burden of CVD [4]. There were approximately 20.5 million deaths in 2021 from CVD [5], and it is estimated that this death toll will soar to 23.5 million by 2030 [6]. This pathology constitutes 7.6% and 21.0% of national healthcare costs, with hospital inpatient care and pharmacological treatment being the largest contributors [7].

Various synthetic drugs are available in the market to treat cardiovascular disorders, including angiotensin-converting enzyme (ACE) inhibitors. It is the latest advancement for treating hypertension, with captopril being the first mediator. Some generic ACE inhibitor tablets may cause side effects like low blood pressure, hives, angioedema, hyperkalemia, dizziness and headaches [8]. Similarly, Clopidogrel is the antiplatelet agent used for coronary artery disease, but when used concurrently with other anticoagulants, it is associated with increased bleeding risk [9]. Statins are another example of synthetic medications that are used in coronary artery disease but have adverse effects like myopathy, rhabdomyolysis, and a transient increase in liver enzyme activity [10,11].

Polyphenols are nature-derived phytochemicals present in medicinal plants, with an enormous structure and functions [12,13]. Polyphenols possess antioxidants, anti-inflammatory, antiplatelet, and immunomodulatory properties that are responsible for the treatment and prevention of cardiovascular disorders [14,15,16]. Existing studies have shown that polyphenols can enhance plasma lipid profiles and endothelial function, decrease the inflammatory responses, and inhibit platelet aggregation, thus providing cardioprotective premises in several ways [17]. Furthermore, the presence of phenolic compounds helps in promoting cardiovascular benefits through endothelial-dependent nitric oxide and insulin-mediated cell signaling pathways, leading to control of lipid and blood sugar levels [18]. Therefore, exploration of more polyphenols with therapeutic potential in cardiovascular diseases from agricultural waste is required.

*Citrullus lanatus* (*C. lanatus*), also called watermelon, is an important plant of the Cucurbitaceae family. The estimated yield of *C. lanatus* is approximately 90 million tons/annum and is ranked in the top 10 fruit crops cultivated worldwide [19]. *C. lanatus* has many health-promoting bioactive compounds, including polyphenols, carotenoids, etc. Furthermore, *C. lanatus* also contained amino acids such as citrulline, arginine and glutathione [20]. The rind of *C. lanatus,* which is about 30–40% of the total fruit weight, is considered agricultural waste. But it is considered a rich source of polyphenols and other valuable phytochemicals [21]. Therefore, the present study was designed to extract polyphenols, including phenolic acids and flavonoids, from the rind of *C. lanatus* and to evaluate their antioxidant potential using a range of in vitro assays. High-performance liquid chromatography (HPLC) was employed to identify and quantify the major bioactive compounds present in the extract. Furthermore, the purpose of the study was to explore the cardioprotective effects of the polyphenol-rich ethanolic extract of *C. lanatus* in a doxorubicin-induced cardiotoxicity model in rats, thereby establishing a link between its polyphenol composition and therapeutic potential.

## 2. Materials and Methods

### 2.1. Reference Standards, Reagents and Chemicals

Chromatography grade solvents, standards and reagents, used in the present study, were procured from Sigma Chemical Co., (St. Louis, MO, USA). Whereas the chemicals for in vitro analysis, including salts, were procured from Merck (Darmstadt, Germany). For in vivo analysis, the kits were used that were procured from China (Nanjing Jiancheng, Bioengineering Institute, Nanjing, China). Doxorubicin (DOX) was obtained as doxorubicin hydrochloride (2 mg/mL) from Citi Pharma, Lahore, Pakistan.

### 2.2. Collection and Pretreatment of Plant Material

The fruit part of the *Citrullus lanatus* was collected during summer (May 2024) from the controlled agricultural fields of Ayub Agriculture Research Institute (AARI, Faisalabad, Pakistan). The sample was also verified by a Taxonomist in the Department of Botany, Government College University Faisalabad (GCUF), Pakistan. The rind of the fruit was detached from the pulp and dried in a hot-air dehydrator and passed through a grinder (LG BL 999SP, Frankfurt, Germany) to produce a fine powder (80-mesh). The resulting powdered sample was stored in a dark place at room temperature in an airtight labeled polythene bag.

The fruit part of the *Citrullus lanatus* was collected during summer (May 2024) from the controlled agricultural fields of Ayub Agriculture Research Institute (AARI, Faisalabad, Pakistan). The sample was also verified by a Taxonomist in the Department of Botany, Government College University Faisalabad (GCUF), Pakistan. The rind of the fruit was detached from the pulp and dried in a hot-air dehydrator and passed through a grinder (LG BL 999SP, Frankfurt, Germany) to produce a fine powder (80-mesh). The resulting powdered sample was stored in a dark place at room temperature in an airtight labeled polythene bag.

### 2.3. Preparation of Extract

A Soxhlet unit was used to extract the powdered sample (300 g) using 700 mL of ethanol solvent, as previously reported by [22]. The extracted material was filtered through Whatman filter paper, and the solvent from the extract was evaporated under reduced pressure. The dried extract was stored in a refrigerator for further analysis, and the yield was calculated as:

$$Yield\left(\frac{g}{100\;g}\right)=\frac{weight\;of\;the\;extract(g)}{weight\;of\;dry\;plant\;material(g)}\times 100$$

The ethanolic extract of Citrullus lanatus was solubilized in 10% DMSO and distilled water (1:9 *v*/*v*) with gentle vertexing and sonication to ensure complete dissolution before experimental use.

### 2.4. Quantification of Phenolic Acids and Flavonoids on HPLC

Analysis of the polyphenol compounds of the rind of *Citrullus lanatus* was carried out on an Agilent 1260 Infinity II liquid chromatography (LC) system (Agilent Technologies, Santa Clara, CA, USA) using a reported method [23]. High Performance Liquid Chromatograph is coupled with an autosampler (G7129A), degasser, binary gradient pump (G7112B), column oven, and multi-wavelength UV/Vis detector (MWD, G7165A). Separation was achieved on a Poroshell 120 C_18_ column (150 × 4.6 mm, 2.7 µm particle size) maintained at 30 °C, with a constant flow rate of 1.0 mL/min. A 10 µL aliquot of each extract was injected into the system. A non-linear gradient elution was applied using a mobile phase consisting of acetonitrile: methanol (70:30, *v*/*v*) and water containing 0.1% formic acid, with the organic phase varying from 15% to 80% over the run. The MWD was set to monitor at multiple wavelengths (250, 270, 290, 310, 330, 350, and 370 nm) with a spectral resolution of 1.2 nm and a data acquisition rate of 10 points per second. Data processing was performed using Agilent OpenLab Data Analysis software (Build 2.204.0.661). Compound identification was based on retention time matching and co-injection with authentic standards, while quantification was carried out using the standard addition method, employing calibration curves derived from the linear relationship between concentration and peak area.

### 2.5. Antioxidant Assay (In Vitro)

#### 2.5.1. Spectrophotometric Determination of Total Phenolic (TP), Total Flavonoid (TF) and Total Flavonol (FOL) Contents of PRCL Rind Extract

The TP and TF contents of the PRCL rind extract were determined as described previously [24]. Total phenolics (TP) were calculated from a standard curve of gallic acid solutions ranging from 10 to 80 µg/mL, and the results were obtained using the equation:

y = 6.2507x + 0.0344; R^2^ = 0.999

The concentrations of phenolics were expressed as mg/g of plant materials, reported on the basis of gallic acid equivalents. Similarly, for the determination of TF contents, a standard curve was prepared using catechin solutions ranging from 10 to 160 ppm. The results were calculated using the following equation, as flavonoids are reported as mg/g of plant materials, based on catechin equivalents:

y = 2.3727x + 0.0048; R^2^ = 0.9986

The flavonol content from the PRCL rind extract was assessed using a previously reported method with slight modifications, as previously described [24]. The 10 mg of extract was dispensed with 1 mL of 2% AlCl_3_ (*w*/*v*) and 1.5 mL of 5% solution of sodium acetate (*w*/*v*). The mixture was then incubated for 2 h at 30 °C. The absorbance was recorded at 440 nm using a UV/Visible Spectrophotometer for both the extract solution and a rutin standard solution (0.025–0.15 mg/mL). The flavonol (FOL) content was quantified with the help of a rutin calibration curve.

y = 8.1339x − 0.0014 with R^2^ = 0.9991

and results were expressed as mg/g as rutin equivalent on the basis of dry plant materials.

#### 2.5.2. Radical Scavenging Assay

The DPPH free radical scavenging activity of PRCL rind extract was evaluated [22]. Initially, the various concentrations ranging from 100 to 1 µg/ mL of extract were prepared using a serial dilution technique. 1 mL PRCL rind extract solution was mixed with 1 mL of a 90 µM freshly prepared 2,2-diphenyl-1-picrylhydrazyl (DPPH) solution in methanol and incubated at room temperature for 30 min. Absorbance was recorded at 515 nm, and DPPH solution served as the blank, while BHT was used as the positive control. The percentage radical scavenging was calculated using the following formula.

$$\mathrm{DPPH}\;\mathrm{radical}\;\mathrm{scavenging}\;(\%)=\frac{Abs.\;of\;90\;µM\;DPPH\;solution-Abs.\;of\;sample\;solution}{Abs.\;of\;90\;µM\;DPPH\;solution}\;\times 100$$

A graph-plotted percentage scavenging against extract concentration helped to calculate the extract concentration giving 50% scavenging (SC_50_) of DPPH radical.

#### 2.5.3. Reducing Potential of PRCL Rind Extract

The reducing power of PRCL rind extract was evaluated based on the method described [22]. Volumes equivalent to extract concentration 0.625–10 mg were added with 2500 µL of 0.2 M phosphate buffer (pH 6.6) and 2500 µL of 1% potassium ferricyanide in test tubes. The contents of the test tubes were incubated for 25 min at 45 °C, followed by the addition of 2500 µL of 10% tricholoroacetic acid. After centrifugation at 5 °C for 10 min using a refrigerated centrifuge, 5 mL of the top layer was collected, diluted using 5.0 mL of distilled water and 1 mL of 0.1% ferric chloride was added. Thereafter, the absorbance at 700 nm was measured to assess the reducing power of the aqueous ethanolic extract.

#### 2.5.4. Inhibition of Linoleic Acid per Oxidation

The antioxidant activity in terms of inhibition of linoleic acid peroxidation by PRCL rind extract was evaluated as previously reported [25]. An emulsion sample was used as a negative control, while BHT served as a positive control. The absorbance was measured at 500 nm both at the beginning of the experiment and after 180 h. The given formula was used to calculate the percentage inhibition

$$Inhibition\%=100-[\frac{Absorbance\;increase\;in\;sample\;after\;180\;h}{Absorbance\;increase\;in\;control\;after\;180\;h}]\times 100$$

### 2.6. In Vivo Cardio Protective Effect

#### 2.6.1. Animals

The present study utilized 30 male *Wistar Kyoto* rats, weighing between 150 and 170 g, which were obtained from the animal facility of the University of Animal and Veterinary Houses (UVAS), Lahore, Pakistan. The animals were housed individually in sanitized propylene cages (16.0 × 13.5 × 6.25 inch) lined with sterile paddy husk bedding and were acclimatized at 25 ± 2 °C temperature and 30–70% humidity. The study was performed under the guidelines of the Institutional Review Board for Animal Studies.

#### 2.6.2. Experimental Design

**Scheme 1:**
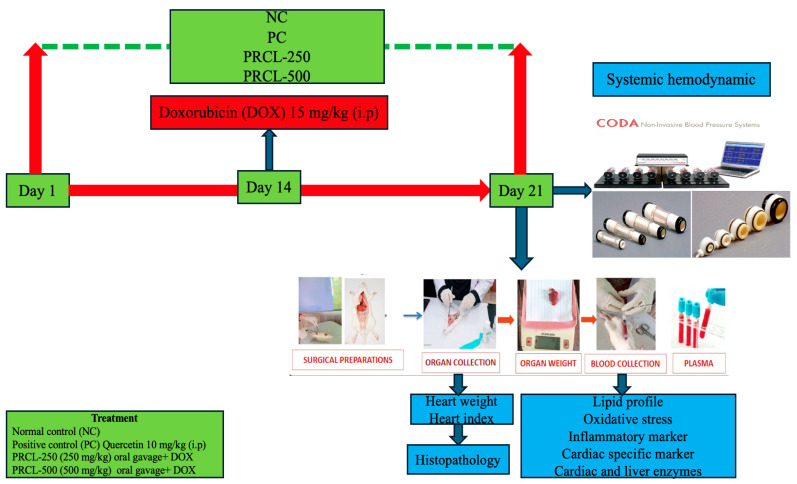
An experiment flow sheet of the design of the experiment for all the groups.

The present study was conducted over a period of 21 days (Scheme 1). After 6–7 days of acclimatization, 30 healthy *Wistar Kyoto* rats were randomly distributed into five different experimental groups, with six animals (*n* = 6) being kept in each group in a separate cage. They were provided with unrestricted access to water. The group allocation was as follows:

Group NC (Normal control group)—Received a standard diet orally (approximately 20 g/rat/day) for 21 consecutive days.

Group DOX (Alone)—Received a single intraperitoneal (i.p.) dose of doxorubicin at 15 mg/kg [26] on day 14 and received a standard diet orally (approximately 20 g/rat/day) for 21 consecutive days.

Group PC (positive control)—Animals treated with Quercetin at a dosage of 10 mg/kg b.w per day for 21 consecutive days and a single intraperitoneal (i.p.) dose of doxorubicin at 15 mg/kg on day 14 and received a standard diet orally (approximately 20 g/rat/day) for 21 consecutive days.

Group-PRCL-250—Animals treated with polyphenols-rich *Citrullus lanatus* rind extract at a dosage of 250 mg/kg Body weight for 21 consecutive days and a single intraperitoneal (i.p.) dose of doxorubicin on day 14 at 15 mg/kg and were given a standard diet orally (roughly 20 g/rat/day) for 21 consecutive days.

Group-PRCL-500—Animals treated with polyphenols-rich *Citrullus lanatus* rind extract at a dosage of 500 mg/kg Body weight for 21 consecutive days and a single intraperitoneal (i.p.) dose of doxorubicin on day 14 at 15 mg/kg and were given a standard diet orally (roughly 20 g/rat/day) for 21 consecutive days.

#### 2.6.3. Model Validation by Measuring Systemic Hemodynamics and Electrocardiogram

On day 21, all animals were trained for non-invasive blood pressure (NIBP) measurement by using a restrainer and tail cuff method by using CODA (Kent Scientific Corporation, Torrington, CT, USA) as reported [27]. After training the animals in the restrainer, the restrainers were putting on a heating pad at level 2 for mild heating to maintain the body temperature, while a cuff was placed around the tail. All procedure was planned and fixed for the number of sub-cycles, the duration of each cycle and the interval between each cycle.

#### 2.6.4. Electrocardiogram (ECG) Recordings Under Anesthesia

One night before day 21, the animals were fasted overnight for 12–14 h, with water available ad libitum. The next day, the rats were anesthetized by intraperitoneal injection of sodium pentobarbitone (60 mg/kg body weight) [28]. An electrocardiogram (ECG) was performed by using lead II on day 21 after taking NIBP, as reported [29,30]. Standard 3-lead surface (left foreleg, left rear leg and right foreleg) ECG recordings were taken by using electrodes connected with gold-plated needles administered under the skin as reported [31]. Recordings were obtained within 2–3 min using a differential amplifier connected to a PowerLab data acquisition unit (ADInstruments, Bella Vista, Australia). The results were averaged for each animal, and these mean values were subsequently pooled to calculate a group average for statistical analysis. R-R interval (s), R-amplitude (mV) and QRS (s) were analyzed on priority on day 21 to validate the doxorubicin-induced cardiotoxicity model.

The retro-orbital puncture procedure was used for collecting blood samples, and serum was separated by centrifugation for 20 min at 3000 rpm for analysis. Subsequently, the rats were decapitated, and the hearts were excised, cleaned of surrounding fat and connective tissue, and rinsed with ice-cold saline. The heart specimens were dried, weighed and then cut into two portions: one half was processed for histopathological examination, and the other half was used for biochemical analysis.

### 2.7. Biochemical Investigations

#### 2.7.1. Lipid Profile

Lipid contents were analyzed from the rat’s serum samples on a semi-automatic analyzer, following the procedure described [32,33]. Briefly, blood samples of the rats were centrifuged; serum was separated and tested for biochemical parameters of lipid profile by a method indirectly, by subtracting the cholesterol content of the infranatant fraction (the sum of the CHDL and CLDL) from the total plasma cholesterol. The phosphotungstate precipitation method is used for the estimation of high-density lipoprotein (HDL) cholesterol. The cholesterol esterase method was used to determine the total cholesterol (TC), whereas the glycerol-3-phosphate oxidase method was used to check the triglycerides (TG). The Friedewald formula was used to determine the low-density and very low-density lipoproteins (LDL and VLDL, respectively) cholesterol levels.

$$\mathrm{VLDL}\text{-}\mathrm{Cholesterol}=\frac{Total\;serum\;triglycerides\;Cholesterol}{5}$$

$$\mathrm{LDL}\text{-}C=\mathrm{Serum}\;\mathrm{cholesterol}-\frac{Total\;serum\;triglycerides\;Cholesterol}{5}-HDL\text{-}C$$

#### 2.7.2. Determination of Lipid-Based Cardiovascular Risk Indices and Cardiac Enzymes

Similarly, Friedewald’s formula [33] is also used for the calculations of all the cardiovascular risk indices, including atherogenic coefficient (AC), atherogenic index of serum (AIS), and cardiac risk ratio (CRR) [34]. The atherogenic index was computed based on the procedure as derived. Atherogenic index was calculated by using the following formula:

$$\mathrm{Atherogenic}\;\mathrm{index}\;=\;\frac{Total\;cholesterol-HDL\text{-}C}{HDL\text{-}C}$$

Cardiac enzymes like cardiac troponin I (cTnI) and lactate dehydrogenates (LDH) were measured from the plasma by using a local pathological laboratory commercial facility. Briefly, Serum levels of cardiac troponin-1 (cTnI) were quantified by using fluorescence immunoassay (ichroma™ Tn-I Plus, Boditech, Chuncheon-si, Gangwon Province, Republic of Korea). Techniques used for the detection of cTnI follow a sandwich immunodetection principle, which allows the attachment of troponin-1 in the sample under study to fluorescence-labeled anti-TnI antibodies, forming antigen–antibody complexes. These antigen–antibody complexes migrate on the strip and are trapped by the immobilized antibodies, which immediately generate a fluorescence signal that is proportional to the levels of cardiac troponin-1 (cTnI) in the sample. Each reaction mixture consists of 50 µL of serum mixed with assay buffer, and a total volume of 75 µL of the mixture was loaded onto the test cartridge. After a short duration of 12 min of incubation, the test cartridge was inserted into the ichroma™ analyzer, and cTnI concentrations were displayed automatically in ng/mL.

Serum LDH activity was quantified using a commercial kinetic UV kit (SPINREACT, S.A./S.A.U., Sant Esteve de Bas, GI, Spain) on an automated chemistry analyzer. The assay is based on the LDH-catalyzed conversion of pyruvate to lactate, accompanied by the oxidation of NADH to NAD^+^. The decline in absorbance of NADH at 340 nm is directly proportional to LDH activity in the sample. Results were expressed in U/L following the manufacturer’s calibration and quality-control guidelines.

#### 2.7.3. Evaluation of Endogenous Antioxidant Levels

The endogenous antioxidant levels were determined from the heart homogenate of rats. Hearts were homogenized in ice-cold tricholoroacetic acid (10%) with 0.05 M phosphate buffer of pH 7.4 and saline. The resulting homogenates were centrifuged at 15,000 rpm for 20 min. These supernatants were used to determine the levels of oxidative stress parameters. Oxidative damage was evaluated by measuring the levels of reduced glutathione (GSH), malondialdehyde (MDA), superoxide dismutase (SOD), glutathione peroxidase (GPx), and catalase following established protocols [35,36]. The activity of superoxide dismutase (SOD) within the cardiac tissues was determined by the method [35]. Briefly, the reaction mixture consisted of xanthine, NBT, and phosphate buffer (pH 7.0). The reaction was initiated by the addition of xanthine oxidase (20 U/mL) and incubated at 25 °C for 20 min. The reaction was stopped with cupric chloride solution, and the absorbance of the formazan product was measured at 560 nm using a spectrophotometer (PowerWave, Bio-Tek Instruments, Winooski, VT, USA).

Lipid peroxidation was quantified by measuring malondialdehyde (MDA) levels using the thiobarbituric acid reactive substances (TBARS) assay. The reaction between MDA and thiobarbituric acid (TBA) forms a colored complex, and absorbance was recorded at 532 nm using a spectrophotometer (PowerWave, Bio-Tek Instruments, USA).

Glutathione peroxidase (GPx) activity was determined by monitoring the oxidation of reduced glutathione (GSH) in the presence of hydrogen peroxide (H_2_O_2_). The decrease in absorbance associated with the conversion of NADPH to NADP^+^ was recorded at 340 nm for 3 min using a spectrophotometer.

Catalase activity was assessed following the procedure described [35]. Briefly, A 10 µL aliquot of tissue lysate (1:10 *v*/*v* dilution with double-distilled water) was mixed with 50 mM phosphate buffer (pH 7.0) to obtain a final volume of 2 mL. The reaction was initiated by adding 1 mL of freshly prepared 30 mM H_2_O_2_, and the decrease in absorbance due to H_2_O_2_ decomposition was recorded at 240 nm using a spectrophotometer.

#### 2.7.4. Estimation of Liver Functions

Liver function was checked using the serum alanine aminotransferase (ALT) and aspartate aminotransferase (AST) tests, and these assays were performed on an auto analyzer as previously reported [37]. Serum was obtained by centrifugation at 3000 rpm for 10 min. These samples were analyzed by using a biochemical autoanalyzer (Opera, Techicon, Bayer, Whippany, NJ, USA) using a protocol based on the enzymatic and colorimetric method.

#### 2.7.5. Measurements of Inflammatory Biomarkers

Tumor Necrosis Factor alpha (TNF-α) and Interleukin-6 (IL-6) in cardiac tissue were quantified using enzyme-linked immunosorbent assay (ELISA) kits for TNF-α (R&D Systems, Inc. Minneapolis, MN, USA) and IL-6 (Abcam Ltd., Cambridge, MA, USA) to evaluate the inflammation level, following the standard protocol [38]. In brief, cardiac tissues were homogenized in a lysis buffer and centrifuged at 13,000 rpm for 20 min at 4 °C to separate the supernatant. The collected supernatant was then diluted with an appropriate buffer, and 100 µL of either the sample or the provided standards was dispensed into the wells of an ELISA plate. The concentrations of TNF-α and IL-6 were measured using commercially available ELISA kits according to the manufacturer’s instructions and the reported method [38]. Results were normalized to tissue weight and expressed as picograms per milligram (pg/mg).

### 2.8. Histopathological Studies

Specimens of heart tissues of each group were fixed in 10% formalin, processed, and embedded in paraffin. Sections of 5 µm thickness were mounted on glass slides, stained with hematoxylin and eosin (H & E) for histopathological examination under a light microscope to assess myocardial injury as previously reported [29,39,40].

### 2.9. Statistical Evaluation

All in vitro experiments were carried out in triplicate and the results are given as mean ± standard deviation. In the case of in vivo studies, six rats were used in each group, and the results are also given in the form of mean ± standard deviation. Statistical comparisons between groups were conducted using one-way Analysis of Variance (ANOVA), followed by Bonferroni or Dunnett (all means) post hoc tests. Analyses were carried out using SPSS 16.0 (IBM, SPSS Inc., Chicago, IL, USA) and Statistica 10.0 (StatSoft Inc., Tulsa, OK, USA). Statistically significant was set at a *p*-value of ≤0.05.

## 3. Results and Discussion

### 3.1. Extract Yield and Polyphenol Composition

The yield (g/100 g) of ethanol extract of *Citrullus lanatus* rinds was found to be 19.58 g/100 g of dry plant material. Table 1 shows the concentration (mg/g) of identified phenolic acids and flavonoids in PRCL rind extract.

**Figure 1:**
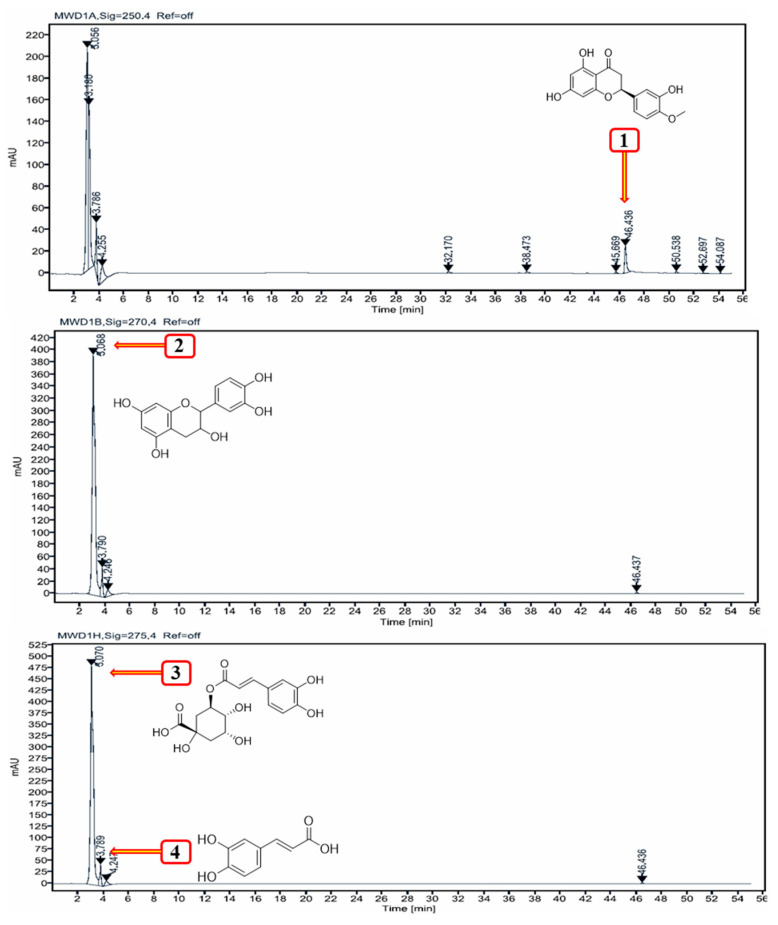
HPLC chromatogram at 250, 270 and 275 nm showing the separation of polyphenols from PRCL rind extract.

Table 1 shows the amount (mg/g) of two phenolic acids, including chlorogenic acid (**3**) and caffeic acid (**4**), and two flavonoids, including catechin and hesperetin, in PRCL rind extract. The caffeic acid (**4**) was found at 3.8 min (0.95 mg/g of dry plant material). In the PRCL rind extract, flavonoids were found in lower concentrations compared to phenolic acids at all the wavelengths studied (Figure 1). Among the flavonoids, hesperetin (**1**) was the most abundant (9.60 mg/g dry weight), followed by catechin (**2**) with 7.18 mg/g dry weight.

Polyphenols are polar compounds and have a strong affinity for ethanol and methanol [45]. Among them, ethanol solvent is generally preferred for recovering food-grade bioactive compounds and is widely favored for antioxidant extraction because of its non-toxic and environmentally friendly nature, as well as its high extraction efficiency [45,46].

### 3.2. Antioxidant Activity (In Vitro)

#### 3.2.1. Total Phenolic, Flavonoid and Flavonol Content

The total phenolic content (TPC) and total flavonoid content (TFC) of the PRCL rind extract were quantified using the Folin–Ciocalteu and aluminum chloride colorimetric methods, respectively. Results were expressed as milligrams per gram of dry plant material in terms of gallic acid equivalents (GAE) for TPC and catechin equivalents (CE) for TFC, as presented in Table 2. The PRCL rind extract exhibited a TPC of 35.6 mg GAE/g and a TFC of 12.8 mg CE/g of dry material. Additionally, the ethanol extract was found to contain 17.6 mg/g of flavonols, expressed as rutin equivalents (RE), also shown in Table 2.

Polyphenols are bioactive compounds widely found in fruits, vegetables, and herbs, and they play a key role in the antioxidant activity of plant materials by functioning as free radical scavengers [47]. Therefore, it is not only beneficial to determine the amount of phenolic content, but also to estimate its relative contribution to the overall antioxidant activity [23]. The total phenolic content is commonly assessed using the Folin–Ciocalteu reagent, depending on the chemical structure of phenolic compounds—specifically, a higher number of hydroxyl (-OH) groups corresponds to a higher phenolic content. Although this method has faced considerable scrutiny and many researchers have highlighted its limitations. This assay is not suitable for comparing samples from different botanical sources due to possible interference from non-phenolic compounds and their matrix components. However, for samples of similar botanical origin, the method remains highly effective due to its simplicity, affordability, robustness, high throughput, and strong correlation with other antioxidant assays, including chromatographic techniques [48].

#### 3.2.2. Free Radical Scavenging Activity

Table 2 shows the DPPH free radical scavenging activity of PRCL rind extract and BHT solution. In comparison to the synthetic antioxidant BHT (RC_50_ = 6.10 µg/mL), PRCL rind extract exhibited lower radical scavenging activity (RC_50_ = 13.6 µg/mL). The free radical scavenging assay assesses an extract’s ability to neutralize radicals using a colorimetric method. This study used the stable DPPH radical, which changes color from violet to yellow when reduced by plant phenolics acting as hydrogen donors [45,49]. The extent of this color change indicates antioxidant activity, which is directly related to the phenolic content and hydroxylation degree. Lipid oxidation, a major concern in the food industry and human health, is driven by free radicals. Antioxidant compounds counteract this by scavenging or neutralizing free radicals, with one key mechanism being the inhibition of oxidation initiation through neutralization of reactive oxygen species [45].

#### 3.2.3. Reducing Potential of PRCL Rind Extract

**Figure 2:**
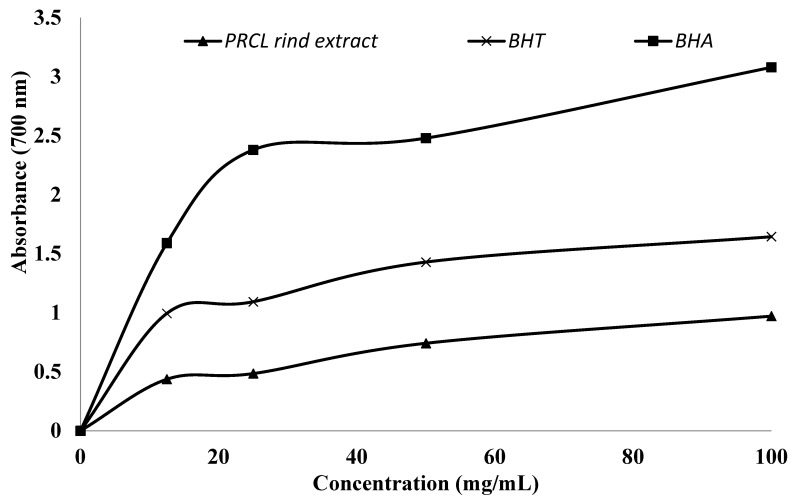
Reducing the potential of polyphenol-rich *Citrullus lanatus* rind extract and synthetic antioxidants.

The trend of reducing the potential of PRCL rind extract was evaluated using a simple colorimetric method and is presented in Figure 2. The Figure shows a positive correlation between absorbance and both antioxidant activity and reducing power of the sample. The present study revealed that the reducing power of the extract was proportional to their extract concentration (12.5 to 100 mg/mL) and expressed high activity at 100 mg/mL. The ethanol extract of *Citrullus lanatus* exhibited absorbance values of 0.43, 0.48, 0.74, and 0.97 at concentrations of 12.5, 25, 50, and 100 mg, respectively. However, its reducing power was considerably lower than that of the standard antioxidants BHT and BHA.

The reducing power assay is based on the capacity of antioxidant extracts or compounds to transfer electrons or hydrogen atoms through their redox potential. The reducing power of phytochemicals in plant extracts is directly proportional to their antioxidant activity. Therefore, evaluating the reducing power assay offers an additional approach to assess antioxidant potential [45]. The reducing potential of antioxidant components is closely linked to their TPC, with extracts containing higher phenolic levels exhibiting greater reducing capacity [50].

#### 3.2.4. Inhibition of Linoleic Acid Peroxidation

The inhibition of linoleic acid peroxidation was also utilized to evaluate the antioxidant activity of PRCL rind extract, expressed as the percentage inhibition of lipid peroxidation, as shown in Table 2. The present study revealed that the PRCL rind extract exhibited 86.9% inhibition of linoleic acid peroxidation, whereas synthetic antioxidant BHT exhibited relatively strong activity, i.e., 89.3%.

The polyunsaturated fatty acids, such as linoleic acid, generate peroxides upon oxidation that could oxidize Fe^2+^ to Fe^3+^, which then form a complex with thiocyanate (SCN^−^). A higher concentration of peroxides in the reaction mixture leads to an increased absorbance, indicating lower antioxidant activity [45]. A study investigated how the total phenolic content in fruits and vegetables relates to their ability to inhibit peroxidation in the linoleic acid system. Their results revealed a positive correlation, indicating that higher levels of phenolic compounds in these foods are associated with greater effectiveness in preventing peroxidation [51].

### 3.3. In Vivo Cardioprotective Effect

#### 3.3.1. Changes in Body Weight and Model Validation

The changes in the body weight, heart weight and percentage increase in body weight and heart indices are shown in Table 3. The present study revealed that the body weight and the percentage increase in body weight of animals treated by DOX (15 mg/kg) were less than the NC group. Administration of PRCL, quercetin and doxorubicin to the rats led to an increase in the percentage increase in body weight in comparison with the DOX-treated group alone. At the same time, heart weight and heart indices were increased in the DOX (15 mg/kg) group when the same was compared to the NC group. Administration of PRCL and quercetin reduced the heart weight when the same were compared to the DOX (15 mg/kg) group, as shown in Table 3.

##### Changes in Systemic Hemodynamics and Electrocardiogram (ECG)

Data related to systolic blood pressure (SBP), mean arterial pressure (MAP) and heart rates (HR) and ECG changes in R-R intervals, R-amplitude and QRS complex are shown in Table 4. The SBP, MAP and HR were decreased in the DOX (15 mg/kg) group when same were compared to the NC group. Administration of PRCL-250, PRCL-500 and quercetin improved the SBP, MAP and HR when the same were compared to the DOX (15 mg/kg) group, as shown in Table 4. This drop in systemic hemodynamics can be associated with the cardiac muscle damage, increased oxidative stress and myocardial injury in the doxorubicin-induced model of cardiotoxicity.

ECG recording have validated the physical indices data, systemic hemodynamic data by showing that treatment with DOX (15 mg/kg) increased R-R interval, R-amplitude and QRS complex when same was compared to NC group while treatment with PRCL-250, PRCL-500 and quercetin reduced the R-R interval, QRS complex and increased R-amplitude when same was compared to DOX (15 mg/kg).

These findings of ECG in the doxorubicin group are similar to previously reported data [52] which showed increased R-R interval, QRS complex and reduced R-amplitude, indicating the establishment of the model. Treatment with polyphenol-rich *Citrullus lanatus* rind extract reverses these abnormality changes in a dose-dependent manner, as shown in Table 4.

The quercetin-treated (positive control) group exhibited cardiac parameters (heart weight, SBP, MAP, HR, RR interval, and ECG parameters) that were comparable to those of the normal control group. This similarity indicates that quercetin maintained normal cardiac physiology through its antioxidant and membrane-stabilizing properties, thereby preventing any deviation from baseline values rather than producing changes higher than the normal physiological levels. These findings are in cohesion with previous findings on the cardioprotective role of quercetin in maintaining myocardial integrity and preserving rather than modifying normal physiological parameters [53,54,55].

#### 3.3.2. Effect of Treatment on Serum Lipid Profile Levels

**Figure 3:**
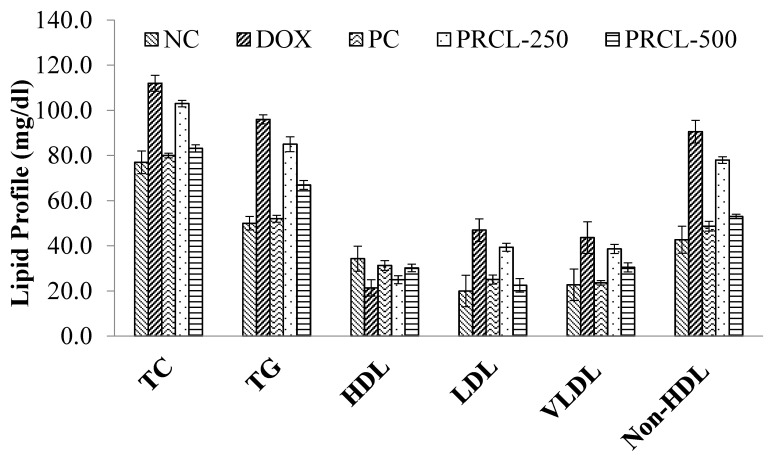
Effect of treatment on the lipid profile of various rat groups. Data are expressed as mean ± standard deviation for six rats per group. Identical superscript letters within the same column indicate no significant differences (*p* > 0.05) among the treatment and control groups. TC, “total cholesterol”; LDL, “low-density lipoprotein”; HDL, “high-density lipoprotein”; TG, triglycerides; VLDL, very low-density lipoprotein, NC: normal control group; DOX, Doxorubicin control; PC, positive control; PRCL-250: Polyphenol-rich Citrullus lanatus rind extract (250 mg/kg Body weight); PRCL-500: Polyphenol-rich Citrullus lanatus rind extract (500 mg/kg Body weight).

Figure 3 shows the effect of PRCL rind extract on serum biochemical markers (Lipid profile levels) in WKY rats. Compared with NC control group, it is clear evidence from the results that doxorubicin (DOX) group remarkably enhanced the serum levels of TC (112.0 mg/dL), TG (96.0 mg/dL), HDL (21.4 mg/dL), LDL (47.0 mg/dL), VLDL (43.6 mg/dL) and non-HDL (90.60 mg/dL) levels in rats and had reduced levels of HDL (21.4 mg/dL). The maximum cardioprotective effect of PRCL rind extract was achieved at the dose of 500 mg/kg and TC, TG, LDL, HDL, VLDL and non-HDL of PRCL-500 group were found to be 83.2, 67.0, 30.2, 22.5, 30.5 and 53.0 mg/dL, respectively. However, treatment with 250 mg/kg of PRCL rind extract also resulted in improvement in some parameters and TC, TG, LDL, HDL, VLDL and non-HDL of PRCL-250 group were found to be 103, 85.0, 25.0, 39.4, 38.6, 78.0 mg/dL, respectively. Overall, the PC control group showed a more protective effect than the PRCL-500 group.

DOX (15 mg/kg) led to a significant decrease in cardiac levels of HDL-cholesterol, while it increased TC, TG, LDL, VLDL, non-HDL levels. In contrast, treatment with the PRCL rind extract resulted in a notable and dose-dependent rise in HDL-cholesterol. The decrease in cardiac HDL in a DOX control group may be linked to suppressed hepatic gluconeogenesis [56].

#### 3.3.3. Effect on Cardiac Enzymes and Lipid-Based Cardiovascular Risk Indices

The effect of PRCL rind extract on cardiac enzymes like cardiac troponin I, lactate dehydrogenate (LDH), lipid-based cardiovascular risk indices like coronary risk ratio (CRR), atherogenic index of serum (AIC) and atherogenic coefficient (AC) of each treatment and control group was assessed and presented in Table 5.

Cardiac troponin and LDH levels were statistically significant (*p* ≤ 0.05) increased 7.5-fold and 2.4-fold, respectively, when compared to their levels in the NC group, as shown in Table 5. Treatment with PRCL-250, PRCL-500 has significantly (*p* ≤ 0.05) reduced these elevated cardiac enzymes when compared to the DOX group, while the maximum response was observed at the highest dose, PRCL-500, as shown in Table 5. Increased LDH in the present study is in line with previously reported data, where Doxorubicin administration increased the LDH levels [57] and troponin I in the serum [58]. Both elevated LDH and troponin I in the current study indicate the loss of myocardial integrity, myocyte membrane rupture, and cardiomyocyte injury, confirming the establishment of the disease model. Treatment with PRCL rind extract normalized the levels of cardiac enzymes in a dose-dependent manner, as shown in Table 5. A similar type of cardiac protection is reported in the literature in reference to *Nigella Sativa* [58] and *Rheum turkestanicum* [59] which justifies the protective role of PRCL extract.

The present study revealed a statistically significant (*p* ≤ 0.05) reduction in lipid-based cardiovascular risk indices in all treatment groups compared to the DOX group. The CRR ratio in the rats of the DOX group (10 mg/kg) was significantly higher than the NC (2.20 mg/dL) group. All treatment groups, along with the PC group, showed a significant reduction in the elevated levels of CRR. Notably, the higher dose (500 mg/kg) of PRCL rind extract produced a more pronounced effect than the lower dose (250 mg/kg) of PRCL rind extract, which was comparable to the PC (quercetin) group (2.50 mg/dL). The AIC of the DOX group was found to be 0.12 mg/dL, which shows a significant increase over the NC group (0.06). The PRCL-500 and PC groups exhibited enhanced protective effects, with atherogenic index of serum (AIS) levels reduced to 0.07 mg/dL in both groups. No significant difference was observed in AIS values between these two groups. In contrast, the doxorubicin control (DOX group) group showed a marked increase in the atherogenic coefficient (AC), reaching 3.93 mg/dL, compared to 1.40 mg/dL in the normal control (NC) group. Notably, the PRCL-500 and PC groups demonstrated a significant reduction in AC values, with the PRCL-500 group recording 1.62 mg/dL—closely matching the PC group at 1.61 mg/dL—indicating a comparable cardioprotective effect. Overall, the increased dose of PRCL rind extract demonstrated more protective efficacy compared to the lower dose.

Atherogenic indices (AI) are powerful indicators of heart disease risk, with higher values indicating an increased risk of developing cardiovascular diseases (CVD), and lower values offering protection against coronary heart disease. This study further emphasizes the cardio-protective potential of the ethanol extract of *Citrullus lanatus* fruit, which demonstrated a dose-dependent reduction in atherogenic indices [60]. A study reported the effect of ethanol extract of *Citrullus lanatus* on cardio-specific biomarkers in an increasing dose-dependent manner from 100 mg/kg to 800 mg/kg, thus showing the cardioprotective effect [60].

Doxorubicin administration markedly elevated plasma troponin I, LDH, and lipid-associated cardiac risk indices (CRR, AIS and AC), reflecting myocardial and metabolic injury. PRCL treatment, particularly at 500 mg/kg, significantly lowered these parameters, indicating protection of cardiac tissue and improvement in lipid homeostasis. These results highlight the cardioprotective and lipid-stabilizing potential of PRCL against Dox-induced toxicity.

#### 3.3.4. Impact on Liver Enzyme Serum Levels

The PRCL rind extract effect on the liver biomarkers of various groups of rats is presented in Table 6. Administration of doxorubicin (15 mg/kg) significantly increased serum ALT (89.4 u/L) and AST (110.3 u/L) levels in the DOX control group compared to those of the NC group, that is, 69.3 u/L and 63.1 u/L, respectively, whereas other treatment groups significantly decreased the levels of ALT and AST. The ALT and AST levels of PRCL-500 and PRCL-250 and PC groups were 76.7, 72.6; 78.3, 86.7; 69.5, 63.8, respectively. Both PRCL rind extract groups lowered the elevated levels of ALT and AST, thus showing the protective effect. Among these, the PRCL-500 group showed more protective effect as compared to a lower dose of PRCL rind extract (Table 6). Overall, the PC group showed the best effect, followed by the PRCL-500 group, when compared to the DOX group.

Aspartate transaminase (AST) and alanine transaminase (ALT) are widely recognized as key biomarkers for detecting potential toxicity. Generally, liver cell damage leads to elevated levels of these enzymes in the bloodstream. Moreover, fluctuations in their levels are indicative of heart damage, like changes seen during liver injury or disease [56]. In the present study, administration of PRCL extract alone (500 mg/kg) did not produce any significant alterations in serum ALT or AST levels compared with the control group, indicating an absence of hepatotoxicity. This suggests that PRCL is well-tolerated and safe at the tested dose. Therefore, the elevated liver enzyme levels observed in the doxorubicin-treated rats are more likely attributed to doxorubicin-induced hepatic stress rather than any adverse effect of PRCL. Increased levels of AST and ALT are reported in previously reported studies where vitamins provide a hepatoprotective role in doxorubicin-induced hepatotoxicity by antioxidant mechanisms [61] and numerous studies reported increased hepatoxicity in the doxorubicin-induced model of cardiotoxicity [62,63]. These findings are presented in Supplementary Table S1 and further support the safety profile of PRCL. This suggests that elevated levels of both liver enzymes might be due to systemic effects of Doxorubicin, which may be involved in mitochondrial injury, increasing membrane permeability, allowing the leakage of intracellular enzymes. Both ROS generation and mitochondrial damage trigger cell death in hepatocytes. Additionally, the extent of enzyme level normalization was lower in the PRCL-250 compared to the PRCL-500 group, indicating a dose-dependent effect. These findings point to potential cardiac damage in rats induced by doxorubicin.

#### 3.3.5. Effect on Oxidative Stress Parameters

Different parameters of oxidative stress were assessed, including SOD, MDA, GSH, GPX and CAT levels in cardiac tissue in all the treated and control groups, are shown in Table 7. The present study revealed that SOD (3.24 NU/mL), GSH (3.01 mg/L), GPX (5.23 NU/mL) and CAT (6.38 NU/mL) levels significantly decreased in the doxorubicin DOX control group compared to the NC group, thereby indicating the oxidative stress in the tested animals.

Table 7 shows that administration of 250 and 500 mg/kg polyphenol-rich *C. lanatus* (PRCL) rind extract significantly increased the levels of SOD, GSH, GPX and CAT. Furthermore, the PRCL rind extract administered at 500 mg/kg demonstrated greater therapeutic efficacy compared to both the 250 mg/kg dose and the PC group. Doxorubicin treatment led to a marked elevation in cardiac malondialdehyde (MDA) levels. As presented in Table 7, all treatment groups, including the PC and both doses of PRCL extract, significantly attenuated lipid peroxidation, resulting in a notable decrease in MDA concentrations relative to the doxorubicin-only group (8.23 nmol/L). The most pronounced reduction in MDA levels (4.01 nmol/L) was observed in the group treated with 500 mg/kg of PRCL. A statistically significant difference was noted between the effects of the two PRCL dosages, with the higher dose exhibiting superior protection against oxidative stress and outperforming the PC group in mitigating lipid peroxidation.

Doxorubicin is an anticancer antibiotic from the anthracycline class, originally isolated in the 1970s from the bacterium *Streptomyces peucetius* var. *caesius* [64]. It is effective against various cancers, including breast cancer, lung carcinoma, and leukemia. However, it is also known for its cardiotoxic effects [64]. Experimental models of cardiotoxicity caused by the chemotherapy drug doxorubicin (DOX) are well-characterized systems [65]. Oxidative stress is considered the most prominent mechanism contributing to the development of DOX-induced cardiotoxicity. The heart is particularly susceptible to this damage due to its low levels of antioxidant enzymes and the increased production of reactive oxygen species (ROS) like superoxide anion and hydroxyl radicals within mitochondria, which induces cardiac injury [66]. These reactive oxygen species damage cell membranes through lipid peroxidation, measured by increased MDA levels, and reduced antioxidant defenses like SOD, GSH, GPx and CAT. Doxorubicin (DOX) generates reactive oxygen species through its quinone structure and interaction with iron, leading to oxidative stress and potential irreversible heart damage [40]. However, treatments like PRCL rind extract that boost antioxidant enzymes may help counteract this oxidative damage. The reduction in GSH levels in rat heart tissue is likely due to elevated lipid peroxidation, which leads to increased GSH consumption [67]. SOD is a key intracellular antioxidant enzyme present in all aerobic cells, where it protects against damage from superoxide anions. It exists in different forms across various cellular compartments, such as CuZn-SOD in the cytosol, Mn-SOD in the mitochondria and EC-SOD in the plasma, allowing for immediate neutralization of superoxide radicals and protecting the cell from oxidative damage. SOD activity can decrease under stress conditions like ischemia or hypoxia [56]. The present study demonstrates that, in addition to these conditions, exposure to doxorubicin also significantly decreases SOD activity. However, treatment with PRCL rind extract at 250 and 500 mg/kg doses resulted in a notable increase in SOD activity. The greater enhancement observed in PRCL-500 compared to PRCL-250 suggests a dose-dependent response to PRCL extract. Catalase activity after injection of doxorubicin is another significant finding in this study, which plays a crucial role in protecting cells by breaking down hydrogen peroxide (H_2_O_2_) into water and oxygen or by acting as peroxidase using H_2_O_2_ as an oxidant. The decreased levels of catalase may be partly due to the suppressed synthesis of this antioxidant enzyme, likely caused by the doxorubicin injection [56]. However, administration of 250 and 500 mg/kg ethanol extract of PRCL resulted in an increase in the respective treatment groups. The elevated GSH levels could be due to the possibility that thiol groups are not the primary targets of doxorubicin-induced cellular injury. The enhanced GSH activity observed in cardiac tissue at higher doses of doxorubicin may reflect an adaptive response, where the antioxidant defense system is upregulated to counteract the increased generation of free radicals caused by doxorubicin exposure [56].

#### 3.3.6. Effect on Inflammatory Biomarkers

The effect of PRCL and Quercetin on inflammatory biomarkers such as TNF-α and IL-6 is given in Table 8. The DOX group showed a maximum level of TNF-α (690.2 pg/mL) and IL-6 (912.6 pg/mL). This is a clear indication of inflammation in this rat group. Reduction in the elevated levels of TNF-α and IL-6 of treatment groups demonstrates the efficacy of the extracts. The greatest reduction in TNF-α (286.8 pg/mL) and IL-6 (311.1 pg/mL) levels was seen in the PRCL-500 group, which is like the PC group.

Doxorubicin induces chronic and severe inflammation in heart tissue by activating the TNF-α signaling pathway, generating excessive reactive oxygen species (ROS), and sustaining the expression of various pro-inflammatory cytokines-contributing to the development of cardiomyopathy [68]. The suppression of pro-inflammatory cytokines and oxidative stress might be the key mechanisms of cardioprotective effect of PRCL rind extract in DOX-induced cardiotoxicity. Additionally, the natural flavonol quercetin has been well-established as a potent antioxidant, known for reducing free radical production and boosting the body’s antioxidant system [38]. Its anti-inflammatory properties have also been demonstrated in both animal and human studies [69].

### 3.4. Histopathology

**Figure 4:**
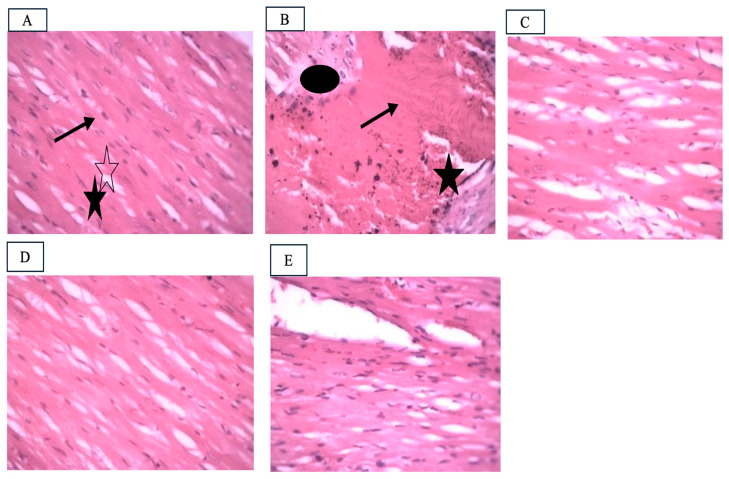
The histopathological microscopic images of the Heart tissue of adult male rats at 40× magnification. (**A**): NC, normal control group; (**B**): DOX, Doxorubicin control; (**C**): PC, positive control; (**D**): PRCL-250, Polyphenol-rich *Citrullus lanatus* rind extract (250 mg/kg Body weight); (**E**): PRCL-500, Polyphenol-rich *Citrullus lanatus* rind extract (500 mg/kg Body weight).

Figure 4A–E shows histopathological investigations of the heart tissue of each group. The morphology of rats in normal control group (A) that were treated with normal food showed healthy cardiac muscle, fine distribution of nuclei’s (arrow) and healthy individual myocytes fibers (star) while DOX group (B) that receive single dose of doxorubicin (15 mg/kg) at day 14 along with normal food showed unbalanced distribution of nuclei’s (arrow) and myocardial fiber necrosis (star) and karyopycnosis with nuclear hyperchromasia (oval). However, administration of PRCL rind extract (250 and 500 mg/kg/day) and quercetin alleviates the doxorubicin-induced histological changes. The higher dose of PRCL rind extract had a remarkable effect than the lower dose. Rats treated with PRCL-500 showed a milder protective effect than the lower dose and showed cardiocyte cytoarchitecture like the control group. Rats treated with PC group showed a moderate protective effect, while rats treated with PRCL-250 showed the least effect. Each group showed a cardioprotective effect. Among all, PRCL-500 showed a remarkable protective effect as indicated by histopathological images.

## 4. Conclusions

The *Citrullus lanatus* rind proved to be a rich source of bioactive polyphenols, yielding 19.58 g extract per 100 g dry material and containing notably high levels of *p*-Hydroxybenzoic acid (163.66 mg/g) alongside lower but relevant concentrations of caffeic acid, catechin, and hesperetin. Although its in vitro antioxidant capacity was a bit lower than that of the synthetic standard BHT, the extract demonstrated dose-dependent reducing power and strong inhibition (86.9%) of linoleic-acid peroxidation. In vivo, the polyphenol-rich *C. lanatus* (PRCL) rind extract markedly attenuated doxorubicin-induced cardiotoxicity: the 500 mg/kg dose normalized serum lipids, decreased atherogenic indices, restored hepatic and cardiac antioxidant enzymes, reduced lipid peroxidation, and lowered pro-inflammatory cytokines to levels comparable with quercetin. Histopathology corroborated these biochemical findings, revealing near-normal cytoarchitecture in the hearts of PRCL-treated rats. Collectively, the data establish *Citrullus lanatus* rind polyphenols, especially at 500 mg/kg, as potent cardioprotective agents that merit further exploration as natural adjuvants against anthracycline-induced cardiac injury. Future studies should focus on isolating individual polyphenolic compounds from *Citrullus lanatus* rind to better understand their specific mechanisms of cardioprotection. Additionally, clinical studies should be undertaken to establish the safety and therapeutic potential of these extracts on human subjects.

## Figures and Tables

**Table 1:** Phenolic profile of polyphenol-rich *C. lanatus* rind extract.

Compounds No.	Compounds Name	RT (Minutes)	*C. lanatus* Rind Extract		References
			Peak Area (%)	Concentration (mg/g of Extract)	
**1**	Hespertin	46.4	257.9	9.60 ± 0.39	[41]
**2**	Catechin	3.0	1377	7.18 ± 0.41	[42]
**3**	Chlorogenic acid	3.1	78	0.38 ± 0.03	[43]
**4**	Caffeic acid	3.8	370	0.95 ± 0.04	[44]

Values are reported as mean ± standard deviation of three independent experiments.

**Table 2:** Extract yield and in vitro antioxidant activity and free radical scavenging capacity of polyphenol-rich *C. lanatus* rind extract.

Antioxidant Assays	Extract	BHT
TPC (mg/g of dry plant material, measured as gallic acid equivalent)	35.6 ± 1.7	---
TFC (mg/g of dry plant material, measured as catechin equivalent)	12.8 ± 0.6	---
TOF (mg/g of dry plant material, measured as rutin equivalent)	17.6 ± 0.5	---
DPPH radical scavenging activity (SC_50_, µg/mL)	13.6 ± 0.7 ^b^	6.10 ± 0.4 ^a^
Inhibition of linoleic acid peroxidation (%)	86.9 ± 3.8 ^a^	89.3 ± 3.3 ^a^

Values are reported as mean ± standard deviation of 3 experiments. Different letters in superscripts in the same row represent significant differences (*p* ≤ 0.05) among the extract and synthetic antioxidant. TPC, total phenolic content; TFC, total flavonoid content; BHT, butylated hydroxytoluene.

**Table 3:** Effect of polyphenol-rich *Citrullus lanatus* rind extract on the body weight, heart weight and heart index of the different groups of cardio-protective rat model on day 21 of the experiment.

Groups	Body Weight (g)		Weight Increase (%)	Heart Weight (gm)	Heart Index (%)
	Initial	Final			
NC	150.5 ± 5	198 ± 5	31.8	0.80 ± 0.002	0.40 ± 0.00
DOX	168.0 ± 5	200.1 ± 6	19.1 *	0.72 ± 0.01	0.36 ± 0.00 *
PC	164.3 ± 6	203.7 ± 5	27.6 #	0.81 ± 0.002 #	0.40 ± 0.00 #
PRCL-250	159.0 ± 5	190.9 ± 6	20.1 #	0.71 ± 0.003 #	0.37 ± 0.00 #
PRCL-500	170.0 ± 6	210.2 ± 7	23.6 #	0.81 ± 0.003 #	0.39 ± 0.00 #

The values are presented as mean ± standard deviation of values of 6 rats. NC: normal control group; DOX: Doxorubicin control group; PC: positive control group; PRCL-250: Polyphenols-rich Citrullus lanatus rind extract (250 mg/kg Body weight/day); PRCL-500: Polyphenols-rich Citrullus lanatus rind extract (500 mg/kg Body weight/day) where * represents in comparison to NC group and # represents in comparison to DOX group.

**Table 4:** Effect of polyphenol-rich *Citrullus lanatus* rind extract on the systolic blood pressure (SBP), mean arterial pressure (MAP), heart rate (HR) and ECG parameters of the different groups of cardio-protective rat model on day 21 of the experiment.

Groups	SBP	MAP	HR	R-R Interval (s)	R-Amplitude (mV)	QRS (s)
NC	132 ± 2	119 ± 1	390 ± 16	0.16 ± 0.002	0.54 ± 0.01	0.017 ± 0.0001
DOX	119 ± 2	93 ± 5 *	360 ± 5 *	0.20 ± 0.001 *	0.45 ± 0.01 *	0.023 ± 0.002 *
PC	130 ± 3	120 ± 1 #	400 ± 10 #	0.16 ± 0.001 #	0.55 ± 0.01 #	0.017 ± 0.0001 #
PRCL-250	122 ± 2	94 ± 1 #	370 ± 5 #	0.19 ± 0.001 #	0.45 ± 0.01 #	0.021 ± 0.0001 #
PRCL-500	128 ± 1	96 ± 1 #	375 ± 5 #	0.18 ± 0.001 #	0.50 ± 0.01 #	0.020 ± 0.0001 #

The values are presented as mean ± standard deviation of values of 6 rats. NC: normal control group; DOX: Doxorubicin control group; PC: positive control group; PRCL-250: Polyphenols-rich Citrullus lanatus rind extract (250 mg/kg Body weight/day); PRCL-500: Polyphenols-rich Citrullus lanatus rind extract (500 mg/kg Body weight/day) where * represents in comparison to NC group and # represents in comparison to DOX group.

**Table 5:** Effect of polyphenol-rich *Citrullus lanatus* rind extract on the cardiac enzyme and cardio-biomarkers of different groups of cardio-protective rat models.

Groups	Cardiac Enzyme		Cardio Specific Markers		
	Troponin I (cTnI) (ng/mL)	LDH (U/L)	CRR	AIS	AC
NC	0.20 ± 0.05 ^#^	130 ± 10 ^#^	2.20 ± 0.25 ^#^	0.06 ± 0.01 ^#^	1.40 ± 0.06 ^#^
DOX	1.50 ± 0.25 *	300 ± 5 *	4.91 ± 0.22 *	0.12 ± 0.01 *	3.93 ± 0.33 *
PC	0.18 ± 0.05 *^#^	110 ± 15 *^#^	2.50 ± 0.13 *^#^	0.07 ± 0.01 ^#^	1.61 ± 0.17 *^#^
PRCL-250	1.10 ± 0.25 *^#^	240 ± 5 *^#^	3.72 ± 0.26 *^#^	0.09 ± 0.01 ^#^	2.33 ± 0.27 *^#^
PRCL-500	0.80 ± 0.25 *^#^	200 ± 5 *^#^	2.60 ± 0.23 *^#^	0.07 ± 0.01 ^#^	1.62 ± 0.23 *^#^

The values are presented as mean ± standard deviation of values of 6 rats of the same group. * Indicates a significant (*p* ≤ 0.05) difference from the NC group; ^#^ indicates a significant (*p* ≤ 0.05) difference from the DOX group. LDH: lactate dehydrogenate; CRR, cardiac risk ratio; AIS, atherogenic index of serum; AC, atherogenic coefficient; NC: normal control group; DOX: Doxorubicin control group; PC: positive control group; PRCL-250: Polyphenol-rich *Citrullus lanatus* rind extract (250 mg/kg Body weight); PRCL-500: Polyphenol-rich *Citrullus lanatus* rind extract (500 mg/kg Body weight).

**Table 6:** Effect of polyphenol-rich *Citrullus lanatus* rind extract on the liver enzymes biomarkers of different groups of cardio-protective rat model.

Groups	Liver Parameters	
	ALT (u/L)	AST (u/L)
NC	69.3 ± 3.2 ^#^	63.1 ± 3.8 ^#^
DOX	89.4 ± 4.2 *	110.3 ± 6.7 *
PC	69.5 ± 3.7 ^#^	63.8 ± 4.0 ^#^
PRCL-250 (DOX + PRCL-250)	78.3.0 ± 2.9 *^#^	86.7 ± 3.3 *^#^
PRCL-500 (DOX + PRCL-500)	76.7 ± 3.3 *^#^	72.6 ± 2.8 *^#^

The values are presented as mean ± standard deviation of values of 6 rats of the same group. * Indicates a significant (*p* ≤ 0.05) difference from the NC group; ^#^ indicates a significant (*p* ≤ 0.05) difference from the DOX group. Abbreviations: ALT, alanine aminotransferase; AST, aspartate aminotransferase. NC: normal control group; DOX: Doxorubicin control group; PC: positive control group; PRCL-250: Polyphenol-rich *Citrullus lanatus* rind extract (250 mg/kg Body weight); PRCL-500: Polyphenol-rich *Citrullus lanatus* rind extract (500 mg/kg Body weight).

**Table 7:** Effect of polyphenol-rich *Citrullus lanatus* rind extract on oxidative stress parameters of different groups of the cardio-protective rat model.

Groups	MDA (nmol/L)	SOD (NU/mL)	GSH (mg/L)	GPX (NU/mL)	CAT (NU/mL)
NC	2.87 ± 0.17 ^#^	6.60 ± 0.30 ^#^	4.67 ± 0.108 ^#^	7.03 ± 0.33 ^#^	9.89 ± 0.50 ^#^
DOX	8.23 ± 0.39 *	3.24 ± 0.27 *	3.01 ± 0.19 *	5.23 ± 0.22 *	6.38 ± 0.34 *
PC	4.12 ± 0.15 ^#^	6.05 ± 0.33 ^#^	4.36 ± 0.22 ^#^	6.44 ± 0.24 *^#^	8.66 ± 0.49 ^#^
PRCL-250	5.61 ± 0.29 *^#^	4.97 ± 0.27 *^#^	3.92 ± 0.12 *	5.98 ± 0.20 *^#^	7.97 ± 0.30 *^#^
PRCL-500	4.01 ± 0.22 *^#^	5.94 ± 0.30 *^#^	4.20 ± 0.11 *	6.71 ± 0.212 *^#^	8.68 ± 0.31 *^#^

All data are expressed as mean ± SD and analyzed using one-way ANOVA, followed by the Bonferroni/Dunnett (all mean) post hoc test. * Represents a significant difference (*p* ≤ 0.05) from the NC group, while ^#^ denotes a significant difference (*p* ≤ 0.05) from the DOX group. NC, WHY normotensive control; DOX, Doxorubicin control; PC, positive control; PRCL-250: Polyphenol-rich *Citrullus lanatus* rind extract (250 mg/kg Body weight); PRCL-500: Polyphenol-rich *Citrullus lanatus* rind extract (500 mg/kg Body weight).

**Table 8:** Effect of polyphenol-rich *Citrullus lanatus* rind extract on anti-inflammatory parameters of different groups of the cardio-protective rat model.

Groups	TNF-α (pg/mL)	IL-6 (pg/mL)
NC	242.3 ± 12.0 ^#^	279.2 ± 12.0 ^#^
DOX	690.2 ± 25.1 *	912.6 ± 40.8 *
PC	272.4 ± 10.0 ^#^	297.8 ± 13.4 ^#^
PRCL-250	399.5 ± 15.6 *^#^	387.9 ± 15.1 *^#^
PRCL-500	286.8 ± 17.0 *^#^	311.1 ± 12.0 *^#^

All data are expressed as mean ± SD and analyzed using one-way ANOVA, followed by the Bonferroni/Dunnett (all mean) post hoc test. * Denotes a significant difference (*p* ≤ 0.05) from the NC group, while ^#^ indicates a significant difference (*p* ≤ 0.05) from the HC group. NC, WHY normotensive control; DOX, Doxorubicin control; PC, positive control; PRCL-250: Polyphenol-rich *Citrullus lanatus* rind extract (250 mg/kg Body weight); PRCL-500: Polyphenol-rich *Citrullus lanatus* rind extract (500 mg/kg Body weight).

## Data Availability

The original contributions presented in this study are included in the article/supplementary material. Further inquiries can be directed to the corresponding author.

## Supplementary Materials

The following supporting information can be downloaded at: https://www.mdpi.com/article/10.3390/pharmaceutics17111469/s1, Table S1: Effect of polyphenol-rich *Citrullus lanatus* rind extract on the liver enzymes biomarkers of different groups of cardio-protective rat model.

## Abbreviations

The following abbreviations are used in this manuscript:

CVD	Cardiovascular disease
PRCL	polyphenol-rich *Citrullus lanatus*
TNF	Tumor necrosis factor
PHBA	*p*-hydroxybenzoic acid
MDA	Malondialdehyde
IL-6	Interleukin 6
