# Supplementary material for: Polyphenol-Rich Citrullus lanatus Rind Extract Mitigates Doxorubicin-Induced Cardiotoxicity: HPLC Profiling and In Vivo Evaluation
Source: Pharmaceutics. 2025 Nov 14;17(11):1469. doi: 10.3390/pharmaceutics17111469 (PMC12655057; doi:10.3390/pharmaceutics17111469)
Supplement: Supplementary file 1 [file pharmaceutics-17-01469-s001.zip › pharmaceutics-3895342-supplementary.pdf]

Supplementary Table 1. Effect of polyphenol-rich *Citrullus lanatus* rind extract on the liver enzymes biomarkers of different groups of cardio-protective rat model

Groups	Liver parameters	
	ALT (u/L)	AST (u/L)
NC	69.3 ± 3.2$^{\#}$	63.1 ± 3.8$^{\#}$
DOX	89.4 ± 4.2*	110.3 ± 6.7*
PC	69.5 ± 3.7$^{\#}$	63.8 ± 4.0$^{\#}$
PRCL-250 (DOX+ PRCL-250)	78.3.0 ± 2.9$^{*\#}$	86.7 ± 3.3$^{*\#}$
PRCL-500 (DOX+ PRCL-500)	76.7 ± 3.3$^{*\#}$	72.6 ± 2.8$^{*\#}$
PRCL-500 (without DOX+ PRCL-500)	69.1 ± 1.7$^{\#}$	63.3 ± 2.0$^{\#}$
